# Capsule Endoscopy in Inflammatory Bowel Disease: Current Status and Issues in Clinical Practice

**DOI:** 10.1002/deo2.70166

**Published:** 2025-06-26

**Authors:** Teppei Omori, Haruka Wada, Tatsuya Mitsui, Mari Hayashida, Noritaka Hibi, Daisuke Saito, Tadakazu Hisamatsu

**Affiliations:** ^1^ Department of Gastroenterology and Hepatology Kyorin University School of Medicine Suginami Hospital Tokyo Japan; ^2^ Department of Gastroenterology and Hepatology Kyorin University School of Medicine Tokyo Japan

**Keywords:** colon capsule endoscopy, inflammatory bowel disease, patency capsule, scoring, small bowel capsule endoscopy

## Abstract

Inflammatory bowel diseases include Crohn's disease (CD) and ulcerative colitis (UC), both of which require endoscopic evaluation of mucosal surfaces. Capsule endoscopy has been used in clinical practice since 2000 as a minimally invasive means of mucosal evaluation. In Japan, there is an innovative algorithm that incorporates capsule endoscopy into the diagnostic algorithm for CD. However, capsule retention is a potential complication, and intestinal patency must be evaluated using a patency capsule or other means before capsule ingestion. In addition, the pathophysiology of CD is a combination of inflammation and stenosis, so the interpretation of score values remains an issue to be addressed. Colon capsule endoscopy for UC is useful in understanding the localization and severity of colorectal inflammation. However, capsule endoscopy is not appropriate for cancer surveillance in patients with UC, and further improvements in bowel preparation are needed. Despite these issues, capsule endoscopy, which allows noninvasive observation of mucosal surfaces, is attractive, and further development is expected.

## 1 Introduction

Inflammatory bowel diseases include Crohn's disease (CD), which mainly involves the small and large intestines, and ulcerative colitis (UC), which involves the large intestine alone. In both diseases, endoscopic evaluation of mucosal surfaces plays an important role not only in diagnosis but also in disease monitoring and evaluating treatment response [1]. Because conventional endoscopy is invasive, alternative means of evaluation such as biomarkers are being investigated. Capsule endoscopy is less invasive than conventional, and is currently being used clinically for intestinal mucosal evaluation. This article aims to review the current status and recent advances in capsule endoscopy in real‐world diagnosis and treatment of CD and UC.

### 1.1 Capsule Endoscopy in CD

CD is a chronic progressive disease of the small and large intestines that damages the intestinal lining via repeated episodes of inflammation. Accumulation of intestinal damage leads to various complications such as stenosis and abscess formation [2, 3], making early diagnosis and appropriate treatment important. Between 70% and 90% of CD patients have small bowel (SB) involvement [4], and 30% have SB involvement alone [5]. In addition, 30% have lesions in the SB that are beyond the reach of ileo‐colonoscopy (IC), which is the gold standard for diagnosing CD [5]. Because these lesions are associated with disease prognosis [6], observation of the entire SB is important, which can be accomplished using SB capsule endoscopy (SBCE) [7].

Leighton et al. demonstrated that SBCE is superior to SB follow‐through (SBFT) and comparable to IC in detecting SB inflammation. In their prospective study of patients with suspected CD of the SB, SBCE combined with IC detected 97.3% of inflammatory lesions, while SBFT combined with IC detected only 57.3% (*p* < 0.001) [8]. SBCE is characterized by high sensitivity but low specificity,[9] and has the potential to detect minor lesions that are undetectable using conventional imaging. In a 2010 meta‐analysis of CD diagnosis, SBCE was superior to IC, radiography, and computed tomography enterography (CTE) and comparable to magnetic resonance enterography (MRE) [10].

Proximal SB lesions are associated with poor clinical outcomes [11, 12] and SBCE is significantly superior to both CTE and MRE in detecting them [13, 14]. SBCE can also identify previously unrecognized lesions and provide an opportunity to optimize treatment. Furthermore, in the prospective Israeli Inflammatory Bowel Disease Research Nucleus study, combining SBCE and MRE resulted in a change in Montreal classification in 64% of cases [15].

SBCE can also reclassify patients with unclassified disease to CD and may be particularly useful for patients with atypical clinical features [16, 17, 18, 19, 20]. Kopylov et al. [21] found that only one‐third of CD patients in clinical and biochemical remission achieved mucosal healing as determined by SBCE. Another study showed that SBCE should not be limited to CD patients with elevated inflammatory markers because the markers are not reliable predictors of SB inflammation [22]. Melmed et al. [23] demonstrated that although the correlation between SBCE and IC mucosal activity score is strong, there is no correlation between clinical/biochemical measures and endoscopic activity score. Therefore, mucosal visualization is important even for CD patients in clinical and biochemical remission.

In a prospective study of CD patients with quiescent disease involving the SB, Ben‐Horin et al. reported a baseline SBCE score of ≥350, the Lewis score (LS), was associated with a 24‐month disease relapse (increase in baseline CDAI > 70, CDAI > 150, treatment intervention) [24].

The same group showed that aggressive treatment modification for high‐risk CD patients (LS ≥ 350) in clinical remission significantly reduced the odds of 24 months of clinical relapse compared with standard therapy (odds ratio [OR], 0.14; 95% confidence interval [CI], 0.04–0.57) [25].

Ukashi et al. also found that LS ≥ 135 in the middle SB segment at baseline caused a composite outcome (bowel surgery, endoscopic dilatation, CD‐related hospitalization, corticosteroid administration, or change in biological/immunomodulatory drug therapy during follow‐up) [26]. A meta‐analysis by Yung et al. in 2018 reported that SBCE, MRE, and bowel ultrasonography can accurately assess recurrence in CD patients who have undergone ileocecal resection; for SBCE, the pooled sensitivity, specificity, diagnostic OR, and area under the receiver operating characteristic curve were 100%, 69%, 30.8 (95% CI, 6.9–138), and 0.94, respectively [27]. In a study of CD patients who underwent intestinal resection, the risks of hospitalization, reoperation, and need for endoscopic dilation were lower in those who underwent follow‐up SBCE than those who did not [28]. In other words, SBCE monitoring beyond the scope of conventional IC may be beneficial in improving postoperative outcomes.

The Japanese guidelines for SB endoscopy state that SBCE is useful for CD diagnosis and follow‐up [7]. The Asia Pacific CD Consensus does not recommend routine SBCE for confirmed CD when there are no clinical symptoms and no obvious findings on MRE or CTE; however, it does recommend SBCE for patients with unexplained anemia or severe malnutrition, or those with a discrepancy between clinical symptoms and other imaging findings [29]. The European Society of Gastrointestinal Endoscopy (ESGE) guidelines have also indicated that SBCE is useful for evaluating and monitoring SB lesion progression in accordance with the “treat‐to‐target” strategy [9].

SBCE is not suitable for all CD patients, as capsule retention within the SB may occur as an adverse event, particularly in those with a long duration of disease and/or SB stenosis. SBCE is clearly not suitable for those with known severe stenosis. To avoid retention, intestinal patency may first be evaluated using a patency capsule before SBCE. Currently, SBCE is best used to screen for SB lesions at the time of diagnosis, in patients with a relatively low degree of intestinal inflammation and damage, and after bowel surgery.

### 1.2 Intestinal Cleansing for SBCE in CD

Performing bowel cleansing may improve the SBCE view and allow for proper mucosal observation Although ingestion of polyethylene glycol (PEG) prior to capsule ingestion optimizes mucosal observation [30], its impact on rates of procedural completion and successful diagnosis has not yet been fully established [31, 32, 33]. In clinical practice, PEG is not commonly used before SBCE. Patients usually adhere to a low‐fiber diet and are instructed to consume a clarified liquid diet the day before surgery and to fast during the 12 h before capsule ingestion. Current ESGE and American College of Gastroenterology guidelines do not address these issues [9, 34].

### 1.3 Patency Capsule

A patency capsule is used to assess bowel patency to avoid SBCE retention. Such use in patients with confirmed CD decreases the SBCE retention rate from 4.63% to 2.88% [35]. Nevertheless, the fact that retention occurs in approximately 3% of SBCE procedures is a major problem. In a retrospective multicenter study on patency capsule safety in Japan [36], which evaluated patients with any disease suspected to cause SB stenosis, the SBCE retention rate was only 0.39%. Furthermore, all cases of retention were due to misjudgment of patency capsule localization during patency evaluation. Therefore, an accurate determination may avoid this adverse event. Failure to obtain a patency assessment in patients with CD using a patency capsule is the only significant predictor of requiring surgical and/or endoscopic dilatation after SBCE, and failure to obtain such an assessment is a significant predictor of worse clinical outcomes [37].

### 1.4 PillCam Crohn's Capsule

The PillCam Crohn's capsule (PCC; Medtronic, Dublin, Ireland), introduced in 2017, was tailored for use in CD patients (it is not yet commercially available in Japan). Its features include a dual‐head capsule with a wide field of view spanning 344°. The system, which includes both hardware and software components, offers a unique approach to assessing mucosal inflammation throughout the small and large intestines. Its software divides the SB into three anatomically equivalent segments based on the length and the colon into right and left portions. Three key parameters are evaluated: lesion distribution, lesion severity, and linear extent [38].

In a study of patients with active CD [39], PCC detected inflammatory lesions in 83.3% of patients, while IC detected lesions in only 69.7%, a difference of 13.6%; moreover, PCC‐identified lesions in 12 patients with normal IC findings. Oliva et al. [40] found that the higher most common lesion score in CD patients undergoing PCC was an independent predictor of the need for intensified treatment (OR, 4.09; 95% CI, 1.80–9.25; *p* = 0.001). In addition, the anatomic extent of disease >30% predicted recurrence (OR, 2.98, 95% CI, 1.26–7.08; *p* = 0.013). In pediatric CD patients, the extent of disease was the only factor associated with recurrence (OR, 4.50; 95% CI, 1.47–13.77, *p* = 0.008). In adult CD patients, lesion severity (expressed as the most common lesion and most severe lesion) was the best predictor of treatment escalation (OR, 4.31; 95% CI, 1.52–12.1, *p* = 0.006).

### 1.5 SBCE Diagnostic Criteria for CD

**FIGURE 1:**
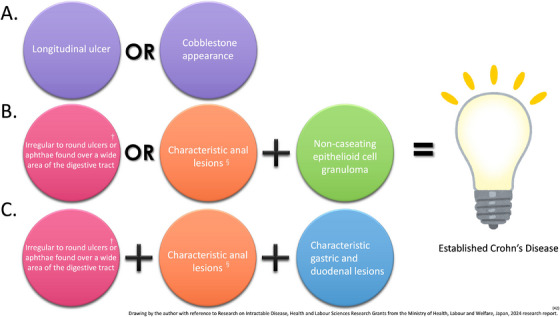
Diagnostic criteria for Crohn's disease in Japan. A definitive diagnosis of Crohn's disease (CD) is made when patterns A, B, or C are met. ^§^Characteristic anal lesions include anal fissures, cavitating ulcers, anal fistulas, perianal abscesses, and edematous skin tags. ^†^lesions must have existed >3 months and involve two or more of the following: esophagus, stomach, small intestine, and colon.

**FIGURE 2:**
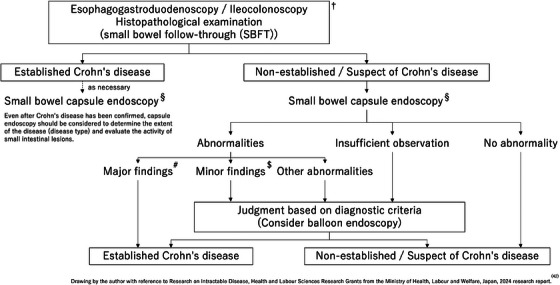
Diagnostic algorithm for Crohn's disease. †Cross‐sectional imaging, abdominal ultrasonography, and biomarkers are also helpful. §performed after confirming gastrointestinal tract patency with a patency capsule. #longitudinal ulcer, cobblestone appearance. $irregular to round ulcers or aphthae over a broad area of the gastrointestinal tract (characterized by a ring of multiple ulcers on the Kerckring fold in the duodenum and small intestine), characteristic gastric or duodenal lesions such as bamboo joint appearance or notch‐like depression.

The diagnosis of CD is usually based on clinical symptoms, inflammatory biomarkers, and endoscopy findings, and confirmed by histologic examination of a biopsy specimen [41]. In Western countries, CD is diagnosed when the SBCE mucosal findings include LS ≥ 135, three or more ulcers, irregular ulcers, and stenosis [16, 17, 18, 19, 20]. In Japan, the diagnostic criteria have been codified by the Ministry of Health, Labour and Welfare's Research Group on Intractable Inflammatory Bowel Disorders (Figure 1) [42]. Basically, the diagnosis is based on morphological findings of IC, barium enema, and SBFT. A diagnostic algorithm is shown in Figure 2. One noteworthy SBCE finding is longitudinal ulcers, which are typically 4 cm or longer along the long axis of the bowel. However, the exact length of the lesion does not need to be considered, as it is difficult to determine the length of the lesion with SBCE findings. Other findings include multiple erosions on the Kerckring fold in the duodenum and small intestine, longitudinal arrangement of erosions, and erosions and ulcers progressing from the jejunum to the ileum [43]. Understanding these characteristic SBCE findings may contribute to the definitive diagnosis of CD. In a prospective study conducted in Japan [44], 15.8% of patients with suspected CD were definitively diagnosed based on SBCE findings. Furthermore, CD lesion detection rates were similar between SBCE and balloon‐assisted enteroscopy, and SBCE was superior to MRE for detecting aphthae and erosions.

### 1.6 SBCE Score in CD

Commonly used SBCE scores for CD include the LS [45] and the Capsule Endoscopy Crohn's Disease Activity Index (CECDAI) (Table 1) [46]. The ESGE recommends using capsule endoscopy scores, such as the LS and CECDAI to assess CD activity over time [9].

**TABLE 1:** Small bowel capsule endoscopy scoring systems for Crohn's disease.

Lewis score (range, 0 ‐ 7840)				CECDAI (range, 0 ‐ 36)		CDACE (range, 0 ‐ 1643)		CE‐CD (range, 0 ‐ 34)＊	
Parameters	Number and characteristics	Longitudinal Range	Morphology	Parameters	Score	Parameters	Score	Parameters	Score
**Villi**	Normal: 0 Edematous: 1	≤10% (short): 8 11%‐50% (long): 12 >50% (whole): 20	Single: 1 Patchy: 14 Diffuse: 17	**A. Inflammation score**	0 = None 1 = Mild to moderate edema/hyperemia/denudation 2 = Severe edema/hyperemia/denudation 3 = Bleeding, erosion, small ulcer (<0.5 cm) 4 = Moderate ulcer (0.5–2 cm), pseudo polyp 5 = Large ulcer (>2 cm)	**Li**. **Inflammatory score**	0 = Normal mucosa 1 = Edematous/reddish appearance 2 = Erosion (<0.5 cm) 3 = Irregular/circular ulcer (0.5–2 cm) 4 = Longitudinal/large ulcer(>2 cm)/cobblestone appearance	**Number of ulcers**	0 = None 1 = 1‐3 2 = 4‐10 3 = ≥11
**Ulcer**	None: 0 Single: 3 Few: 5 Multiple: 10	≤10% (short): 5 11%‐50% (long): 10 >50% (whole): 15	<1/4: 9 1/4‐1/2: 12 > 1/2: 18	**B. Extent of disease**	0 = None 1 = Focal disease (i.e., single segment) 2 = Patchy disease (i.e., multiple segments) 3 = Diffuse disease	**R**. **Range of inflammation**	0 = None 1 = ¼ range 2 = 2/4 ranges 3 = ¾ ranges 4 = 4/4 ranges	**Size of ulcer**	0 = None 1 = Aphtha 2 = <1/4 of the image 3 = >1/4 of the image
								**Extent of disease**	0 = None 1 = ≤10% 2 = 11‐50% 3 = >50%
**Stenosis**	None: 0 Single: 14 Multiple: 20	Ulcerated: 24 Non ulcerated: 2	Traversed: 7 Not traversed: 10	**C. Stricture score**	0 = None 1 = Single passed 2 = Multiple passed 3 = Obstruction	**S**. **Stenosis score**	0 = None 1 = Single passed 2 = Multiple passed 3 = No passage	**Stenosis score**	0 = None 1 = Single passed 2 = Multiple passed 3 = No passage

Lewis score^(^ ^45^ ^)^: Inflammation score most severe tertile: (appearance of villi x range x morphology) + (number of ulcers x range x morphology)] + stenosis score (number x presence/absence of ulcers x traversability).

CECDAI^(^ ^46^ ^)^: The small intestine is divided into proximal (1) and distal (2) segments based on small intestine transit time, and the score is calculated as the sum of the proximal segment score and the distal segment score (A1×B1+C1)+(A2×B2+C2).

CDACE^(^ ^54^ ^)^: The small intestine is divided into quartiles (Li1‐4) using the 25%, 50%, and 75% progress indicators. (Inflammation score: Li1+Li2+Li3+Li4) x 100 + (quantile score: R) x 10 + (stenosis score: S).

CE‐CD^(^ ^56^ ^)^: The small intestine is divided into three parts based on the small intestine transit time. The sum of the ulcer number score + ulcer size score + lesion extent score + stenosis score for each part (*score for children).

The LS grades inflammatory changes in the SB mucosa and was developed to evaluate not only CD but also inflammatory changes caused by nonsteroidal anti‐inflammatory drugs. The three parameters of the LS are edematous changes of villi, ulceration, and stricture [45]. The score ranges from 0 to 7840. A score <135 indicates normal or clinically insignificant inflammatory changes. Mild inflammation is indicated by a score between 135 and 790, A score >790 is considered moderate to severe inflammation. The CECDAI is specific for SB lesions in CD [46]. A CECDAI cutoff value of 3.5 has been reported as a possible predictor of therapeutic intervention [47].

The CECDAI has been validated, and interrater reliability is good [48]. Although the LS and CECDAI have been correlated with each other [49, 50], the equivalent scores have varied depending on the population studied [49, 51, 52, 53]. The CECDAI combines the scores of the two quadrants of the SB, but the LS adds only the highest‐scoring quartile of the three quadrants of the small intestine to the score, making it difficult to reflect the inflammation in the entire small intestine [53]. For this reason, the cumulative LS, which combines the scores of all three quartiles, showed an improved correlation with CECDAI [49]. Also, because the LS is calculated with a higher stenosis score, there is a significant difference in the impact of stenosis between the two scores [53].

The CD Activity in Capsule Endoscopy (CDACE) score focuses more on inflammation by clarifying the inflammation and stenosis components that characterize intestinal lesions in CD [54]. This score ranges from 0 to 1643 and is composed of a location inflammatory score (Li), range score (R), and stenosis score (S). It is calculated as follows: (Li score × 100) + (R score × 10) + S score. The most important feature of the CDACE score is that the first digit reflects the presence or absence of stenosis, which may clarify the pathophysiology of CD, which is a mixture of different elements: inflammation and organic stenosis. The CDACE score correlates well with both the Lewis and CECDAI scores, particularly the latter (*ρ* = 0.915; *p* < 0.0001) [54]. A CDACE score ≥420 is a predictor of therapeutic intervention for SB lesions at 1 year in CD patients with CD activity index score <150 and CRP concentration <0.5 mg/dL [55].

Of the CDACE component values, only the Li score is a continuous variable; the R and S scores are ordinal, so caution should be exercised in their interpretation. However, this score is strongly weighted toward inflammation data, and the R and S scores may not affect the correlation with the other scores. Further validation studies using only the Li score are desirable. However, it is advantageous that the extent of inflammation and degree of stenosis are clearly defined from the viewpoint of understanding the pathophysiology.

The Capsule endoscopy‐CD (CE‐CD) index was proposed by an Italian group as a score for pediatric CD patients [56]. It showed moderate (Pearson's *r* = 0.581, *p* <0.001) and strong (r = 0.909, *p* <0.001) correlation with the Lewis and CECDAI scores, respectively [56]. Its correlation with the CDACE was strong as well (r = 0.965, *p* <0.001) [57]. Comparison of the Pediatric CD activity index (PCDAI) and CE‐CD scores showed that 92% of patients with a PCDAI >30 had a CE‐CD ≥13; however, 26.5% of patients with PCDAI <10 also had CE‐CD ≥13 [56].

Recently, the Capsule Endoscopy Scoring System for CD (CESS‐CD) was developed for the diagnosis of early CD. Defined as age (≥31) (3 points), linear erosion/ulceration (4 points), and circumferential erosion (4 points), with a cutoff value of 7 points, it showed a sensitivity of 85.4%, specificity of 80.0%, positive predictive value of 83.7%, and negative predictive value of 82.1% for CD diagnosis [58].

An international Delphi consensus statement [59] defines an aphthous erosion as “a small loss of epithelial layer with a red halo with normal surrounding mucosa and a white center,” a deep ulcer as “loss of tissue with clear and deep lichen white compared to surrounding swollen and edematous mucosa,” and a superficial ulcer as an ulcer “with mildly sunken tissue loss that does not match the definitions of aphthous erosions and deep ulcers.” In CECDAI and CDACE, the lesion is judged according to lesion size. In contrast, the LS does not mention aphthae or erosions [45]. Recognizing all mucosal defect lesions as ulcers in the LS is also a consideration, but on the other hand, it is controversial because it can be overdiagnostic. Because the presence of small intestinal ulcerative lesions larger than 5 mm affects outcome [60], we consider lesions larger than 5 mm to be ulcers [61].

Based on these considerations, SBCE in CD should be evaluated by a gastroenterologist who understands the endoscopic imaging characteristics of CD and has experience with conventional endoscopy [62].

### 1.7 PCC Score

The PCC score was introduced by Eliakim et al. [63] as a new scoring system using the most common lesion, most severe lesion, extent of disease, and presence of stenosis as components (Table 2). The PCC score proved to be a reliable scoring system with very high interrater agreement (interclass classification coefficient, 0.9; *p* < 0.0001). A moderate correlation was found between PCC score and fecal calprotectin (FC), while the correlation between LS and FC was weak (*r* = 0.54 and 0.32, respectively; both *p* = 0.001) [63]. A recent study [64] further supports the reliability of PCC scores for CD patients with active disease who underwent PCC prior to initiation of biologic therapy. During follow‐up, the PCC score was found to be more responsive to changes in CRP and FC than LS. Furthermore, the PCC score had a better correlation with CDAI than LS [64]. The cumulative nature of the PCC score may make it the preferred scoring system among CD patients undergoing PCC because it better reflects true inflammatory burden than LS.

**TABLE 2:** PillCam Crohn's capsule scoring system.

PillCam Crohn's capsule score [Eliakim score]	
Parameters	Calculation
**A. Most common lesion (MCL)**	None: 0 Mild: 1 Moderate: 2 Severe: 3
**B. Most severe lesion (MSL)**	None: 0 Mild: 1 Moderate: 2 Severe: 3
**C. Anatomic extent of disease**	None: 0 0%–10%: 1 10%–30%: 2 30%–60%: 3 >60%: 4
**D. Stricture**	None: 0 One traversed: 1 >1 traversed: 2 Retention: 3

PillCam Crohn's capsule score [Eliakim score].^(^ ^63^ ^)^

S score (SB1/SB2/SB3/RC/LC) = [(A+B)×C] + D.

SB PCC score = SB1 + SB2 + SB3.

Pan‐enteric PCC score = SB1 + SB2 + SB3 + RC + LC.

PCC, PillCam Crohn's capsule; RC, right colon; SB, small bowel.

### 1.8 Association of Biomarkers With SBCE Findings

SB involvement in CD is not easily reflected in clinical symptoms and cannot be determined by measurement of CRP concentration [21, 44, 60, 65, 66]. However, it is an independent risk factor for relapse and surgery, and monitoring SB involvement is of high clinical significance. Kopylov et al. [21] demonstrated that SBCE has the potential to detect lesions even in patients in clinical and biomarker remission. However, SBCE is an expensive test, and one must decide which patients should be prioritized for SBCE in those without symptoms and without elevated CRP.

FC is the most abundant cytoplasmic protein in neutrophils and plays an important role in inflammation. When the intestine is inflamed, chemokines and cytokines increase the permeability of the intestinal barrier, resulting in neutrophils migrating to sites of inflammation. In turn, FC concentration in stool increases [67].

There is no significant correlation between FC concentration and SB lesion activity and FC concentration has not shown adequate performance for diagnosing SB lesions [68, 69, 70]. In a study of disease activity in CD patients using IC, FC was significantly correlated with CDAI and the simple endoscopic score for CD, but the correlation was lower in Montreal Classification L1‐CD patients than in Montreal Classification L2/3‐CD.[71] Therefore, there is no definitive opinion regarding the usefulness of FC for detecting SB lesions; however, the lack of deep SB evaluation in these studies and the presence of active colorectal lesions may have had an impact. In fact, recent SBCE studies have reported that FC concentration is moderately correlated with LS and CECDAI [49, 51], and a cutoff value of 100 µg/g may be useful in predicting the detection of LS ≥ 135.[72]

In another study, a significant correlation was found between LS and FC concentration, and the sensitivity, specificity, positive predictive value, and negative predictive value of FC >100 µg/g for predicting SB lesions was 80%, 50%, 80%, and 50%, respectively [73]. The sensitivity and specificity were even higher when the FC cutoff value was increased from 100 to 265 µg/g.

Ukashi et al. [74] found that FC concentration increased in conjunction with the expansion of inflammation from the proximal to the distal small intestine, and this change was not observed with CRP. This evaluation of FC as a unique topographic biomarker should lead one to suspect the presence of inflammation in the proximal small intestine with FC concentration ≥77 µg/g, even in patients with no inflammation observed on IC. Furthermore, even in patients with no inflammation in the colon and only mild activity in the distal small intestine, an FC value of ≥234 µg/g may indicate clinically significant inflammation in the proximal SB, which should prompt SBCE.

Recently, leucine‐rich α2‐glycoprotein (LRG) has been used as a biomarker for SB involvement in CD [75, 76, 77]. LRG is one of the acute phase proteins produced not only at the inflammatory site of the intestine but also from the liver by the stimulation of proinflammatory cytokines including tumor necrosis factor‐α, interleukin (IL)‐1β, IL‐6, and IL‐22 [78]. Several studies have reported on the association between SBCE findings and LRG concentration, suggesting that it may be a useful biomarker for predicting SB lesions. LRG concentration is significantly correlated with the LS, CECDAI, and CDACE [79]. LRG ≥14 µg/mL appears to be useful in predicting the presence of ulcerative lesions in the SB larger than 0.5 cm in CD patients with CDAI <150 and CRP <0.5 mg/dL (those in clinical remission) [61]. In addition, an LRG cutoff value of 14 µg/mL predicts SB lesions in patients with LS ≥350 with 100% sensitivity, 80% specificity, 41.7% positive predictive value, 100% negative predictive value, and 82.5% accuracy [80]. The presence of disease activity in CD as determined by LRG has also been demonstrated to be accurate in a recent meta‐analysis [81], with sensitivity and specificity of 77.0% (95% CI, 67.8% to 84.2%) and 81.1% (95% CI, 72.6% to 87.4%). LRG and FC appear to have similar usefulness in detecting endoscopic disease activity in CD [82, 83].

Higher LRG concentrations are more strongly associated with CD‐related hospitalization, need for surgery, and clinical recurrence than lower concentrations [84]. Based on these findings, measurement of LRG in CD patients with SB involvement, even in the absence of clinical symptoms and negative CRP, may be a surrogate marker that triggers SB evaluation.

### 1.9 Capsule Endoscopy in UC

UC is characterized by repeated remissions and relapses [85]. Achievement of mucosal healing improves clinical outcomes and results in fewer bowel resections and steroid‐free clinical remission [5]. For this reason, colonoscopy is the gold standard for evaluating mucosal surfaces in UC patients; however, colonoscopy is invasive and uncomfortable for the patient, so sedation is typically required. As a result, surveillance prevalence is low. Vienne et al. reported that only 54% of patients with inflammatory bowel disease underwent colonoscopy during a 4‐year observation period [86]. In addition, preparations containing high laxative doses decrease the acceptance of colonoscopy in patients with UC [87]. Therefore, a less invasive means of evaluation, such as colon capsule endoscopy (CCE), may fill an unmet need with regard to determining disease severity.

The first‐generation PillCam Colon (CCE‐1) is highly consistent with colonoscopy in terms of determining UC severity (*k* = 0.751, *p* <0.001) and extent of disease (*k* = 0.522, *p* <0.001) on the Baron scale [88]. However, the accuracy of colonoscopy for assessing mucosal healing is low [89, 90]. The CCE‐1 tends to underestimate the extent of disease because of its resolution. The first study on the utility of the second‐generation CCE (CCE‐2) in assessing the severity of mucosal inflammation in UC patients was performed in 2013 [91]. When using a low volume (2 L) of polyethylene glycol and prokinetics (mosapride citrate and metoclopramide), only 69% of CCE‐2 tests could be completed within 8 h, and good or excellent bowel cleansing levels were achieved in less than 50% of patients. However, a strong correlation between CCE‐2 and colonoscopy was demonstrated in the disease activity index (Matts endoscopy score). The correlation values were particularly strong in the cecum, ascending colon, transverse colon, and proximal left colon (r values ranging from 0.765 to 0.906), and moderate in the distal portion of the left colon (*r* = 0.673). In a prospective study [92] comparing the CCE‐2 with the Mayo endoscopic score (MES) and the UC endoscopic index of severity (UCEIS) for assessing mucosal lesions and disease activity, good agreement was found. A satisfaction survey in the same study revealed that 68% of patients preferred CCE‐2 over colonoscopy for disease monitoring, which may influence long‐term compliance; however, the authors noted challenges with respect to cost, test time, and preparation.[92]

Studies of UC in clinical remission (defined as clinical activity index ≤ 4) have reported that the MES and UCEIS scores assessed using the CCE‐2 were predictive of subsequent outcomes [93]. Based on these findings, the CCE‐2 may be used to clinically monitor UC patients. However, although the CCE‐2 can detect polyps larger than 6 mm with a sensitivity of 80% to 95%, biopsy or resection is not possible; therefore, it does not have a role in surveillance for UC‐associated neoplasia [94, 95].

### 1.10 Intestinal Cleansing for CCE in UC

Because UC inflammation is diffuse rather than patchy and can be detected by mucosal CCE‐2 through fecal crevices, the severity of mucosal inflammation may be assessed without the cleaning efficacy that has been advocated for colonic polyp detection. Okabayashi et al. reported a CCE‐2 pretreatment method optimized for UC to achieve better acceptance [96]. In this regimen, 500 mL of hypertonic polyethylene glycol and 250 mL of water are ingested 2.5 h before and 1, 3, and 6 h after capsule ingestion until the capsule endoscope is excreted; castor oil is added at the second intake. A high excretion rate (94%) was obtained, even with a regimen less than the standard CCE pretreatment [4]^−6L^. They also found high interobserver agreement (ĸ = 0.700) for assessing the severity of mucosal inflammation with CCE‐2, even at washout levels that were inappropriate for detecting colonic polyps. Therefore, it is necessary to develop lower formulation doses because higher laxative doses decrease patient acceptance.

### 1.11 CCE Scoring System for UC

The MES and UCEIS are frequently used in clinical practice to assess the severity of UC. However, CCE does not provide the ability to insufflate, wash the bowel during observation, or change the field of view owing to the passive nature of the image. Therefore, the Capsule Scoring of Ulcerative Colitis (CSUC) was developed to evaluate CCE, which is currently the only CCE score used to assess inflammation in UC (Table 3). The correlation between CSUC and clinical parameters (blood tests, FC, and CRP) and Lichtiger's index was similar to that of UCEIS measured by colonoscopy. Furthermore, the CSUC was validated as a predictor of disease relapse in patients in clinical remission [97]. In another study, the CSUC was significantly higher in patients who relapsed within 1 year than in patients who maintained clinical remission (2.83 ± 1.95 vs. 0.72 ± 1.00; *p* < 0.01), and patients with a CSUC ≤1 or within 6 months after achieving induction of remission maintained clinical remission for 1 year [98].

**TABLE 3:** Capsule scoring of ulcerative colitis.

CSUC (range, 0–14)		
Parameters (score most severe lesions)	Calculation	
**Vascular pattern**	Normal: 0 Patchy obliteration: 1 Obliterated: 2	Normal vascular pattern Obliterated area ≤30% Obliterated area ≥30%
**Bleeding**	None: 0 Mild: 1 Severe: 2	No visible blood detected by Suspected blood indicator (SBI) No. of bleeding pictures detected by SBI ≤10 No. of bleeding pictures detected by SBI ≥10
**Erosions and ulcers**	None: 0 Erosions: 1 Superficial ulcer: 2 Deep ulcer: 3	Normal mucosa, no visible erosions or ulcers Tiny (≤5 mm) defects in the mucosa Larger (≥5 mm) defects in the mucosa Larger (≥5 mm) and deeper excavated defects in the mucosa, with a slightly raised edge

Capsule Scoring of Ulcerative Colitis (CSUC).^(^ ^97^ ^)^

Severity scores were scored in the distal and proximal colon regions (divided at the splenic flexure).

Vascular pattern total (proximal + distal) + bleeding total (proximal + distal) + erosions and ulcers in total (proximal + distal) (lowest value–highest value, 0–14).

## 2 Conclusion

This review reported the current status and usefulness of capsule endoscopy in CD and UC patients. In patients with CD, SBCE cannot be used for all, and intestinal patency must be evaluated before its use; interpretation of score values is also an issue. The PCC, which can observe both the small and large intestine and is already in use in Europe and the United States, has yet to be introduced in Japan. In patients with UC, CCE cannot be used for cancer surveillance, and further improvements are needed with respect to bowel preparation. However, the capsule endoscopy system, which allows noninvasive observation of mucosal surfaces, is attractive, and further development is expected.

## Conflicts of Interest

Teppei Omori has received lecture fees from AbbVie GK., Takeda Pharmaceutical Co. Ltd., and KISSEI Co. Ltd. Haruka Wada has no conflicts of interest. Tatsuya Mitsui has no conflicts of interest. Mari Hayashida has no conflicts of interest. Noritaka Hibi has no conflicts of interest. Daisuke Saito has received lecture fees from AbbVie GK, Mitsubishi Tanabe Pharma Corporation, Mochida Pharmaceutical Co., Ltd., Takeda Pharmaceutical Co. Ltd., JIMRO Co., and Janssen Pharmaceutical K.K. Tadakazu Hisamatsu has performed Joint Research with EA Pharma Co. Ltd. and Kissei Pharmaceutical Co. Ltd. He has received grant support from Mitsubishi Tanabe Pharma Corporation, EA Pharma Co. Ltd., AbbVie GK, JIMRO Co. Ltd., Zeria Pharmaceutical Co. Ltd., Kyorin Pharmaceutical Co. Ltd., Nippon Kayaku Co. Ltd., Takeda Pharmaceutical Co. Ltd., Pfizer Inc., Boston Scientific Co. Ltd., and Mochida Pharmaceutical Co., Ltd., and consulting and lecture fees from EA Pharma Co. Ltd., AbbVie GK, Janssen Pharmaceutical K.K., Pfizer Inc., Mitsubishi Tanabe Pharma Corporation, Kyorin Pharmaceutical Co. Ltd., JIMRO Co., Mochida Pharmaceutical Co., Ltd., Gilead Sciences Inc, Bristol Myers Squibb Co. Ltd., Eli Lilly, Abivax, and Takeda Pharmaceutical Co. Ltd.

## Ethics Statement

N/A.

## Consent

N/A.

## Clinical Trial Registration

N/A.
